# Molecular profiling of microinvasive breast cancer microenvironment progression

**DOI:** 10.1186/s12967-019-1936-x

**Published:** 2019-06-03

**Authors:** F. Lessi, C. Scatena, P. Aretini, M. Menicagli, S. Franceschi, A. G. Naccarato, C. M. Mazzanti

**Affiliations:** 1 Genomic Section, Fondazione Pisana per la Scienza ONLUS, via Ferruccio Giovannini, 13, S. Giuliano Terme (PI), 56017 Pisa, Italy; 2 Department of Translational Research and New Technologies in Medicine and Surgery, University of Pisa, Pisa, Italy

**Keywords:** Cancer microenvironment, Breast cancer, Laser capture microdissection, RNA-seq, Cancer progression

## Abstract

**Background:**

Tumors develop by progression through a series of stages. Every cell of the tumor microenvironment is constantly changing in the flow of the cancer progression. It has become clear in recent years that stroma is essential for tumor maintenance and growth. Here, we aimed to give a chronological order of gene expression changes given in the dynamical framework of microinvasive breast cancer microenvironment.

**Methods:**

RNA-seq was performed on seven microinvasive breast cancers. For each of them we microdissected seven different portions of the tumor, four related to the breast epithelium and three to the stroma. Breast epithelium was chronologically subdivided in normal breast epithelium (NBE), carcinoma in situ (CIS), emerging invasive fingers (EIF) and invasive breast cancer (IBC). For each of the breast epithelium subdivisions we collected the adjacent stroma (S): S-NBE, S-EIF and S-IBC.

**Results:**

The overall differentially expressed genes (DEGs) in all the compartments were analysed and evaluated to understand the pathways involved in tumor progression. Then we analysed the DEGs of the epithelial and stromal portions in comparison with the normal portions. We observed that the stromal cells are necessary for the development and the maintenance of the tumor, especially in tumor progression. Moreover the most important genes involved in the main metabolic pathways were analysed and the communications within the different cell compartments were highlighted.

**Conclusions:**

As a future perspective, a deeply study of the identified key genes, particularly in the stromal cells, will be crucial to develop an anticancer therapy that is undergoing a conversion from a cancer cell-centric strategy to a stroma-centric strategy, more genomically stable.

**Electronic supplementary material:**

The online version of this article (10.1186/s12967-019-1936-x) contains supplementary material, which is available to authorized users.

## Background

Tumors develop by progression through a series of stages. It is now widely accepted that cancer is attributed to the accumulation of genetic alterations in cells. Every cell of the tumor microenvironment is constantly changing in the flow of the cancer progression. The possible role of the tumor microenvironment in neoplastic development has been investigated since the late nineteenth century, with studies published by Stefano Paget in 1989 [1]. The structure and functions of the tumor microenvironment, as well as the relationships with the neoplasia, allow to define more precise prognostic and therapeutic directions.

Breast cancer carcinogenesis is well known, characterized by well defined stages, starting from the atypical ductal hyperplasia progressing to ductal carcinoma in situ (DCIS) and ending, although not necessarily, with the invasive breast cancer (IBC). [2].

In breast cancer, epithelial cells require the stroma to meet their needs of nutrition, waste removal, and structure. It has become clear in recent years that stroma is, indeed, essential for tumor maintenance and growth which can also provide protection from the human immune system attacking the cancer cells [3, 4]. The tumor microenvironment is characterized by an increased number of fibroblasts, expressing alpha-smooth muscle actin, so-called cancer associated fibroblasts (CAFs). Therefore it is important to integrate gene expression changes of both tumoral cells and cancer-associated stroma, occurring during the difference phases of tumor progression. For this reason we focused our attention on a specific kind of breast cancer such as the microinvasive breast carcinoma (MIBC), which is a rare entity in which an invasive component not exceeding 1 mm is found, mostly in a DCIS setting [5]. MIBC accounts for about 5–10% of DCIS with a very good overall prognosis for the patients [6]. The peculiar characteristic of this tumor histotype, that meets our needs, is that we are able to identify on the same tumor section at the meantime all phases of breast cancer progression: normal tissue, DCIS and invasive foci with the respective surrounding stroma.

Formalin-fixed, paraffin-embedded (FFPE) tissue samples stored in diagnostic pathology archives represent an invaluable bio-bank for retrospective clinical research. This interest is primarily driven by the fact that the process of creating FFPE tissue is the most common technique used by clinical and/or research pathologists for tissue processing, evaluation, diagnostics, immunoanalysis, preservation, and archiviation. The use of FFPE samples in molecular studies presents some great advantages, for example, these types of samples are available and readily accessible in vast quantities, which is a very important element considering a rare disease such as MIBC. The cost associated with their storage is low, as well, and the significant association between pathological and clinical annotations makes FFPE tissue an attractive specimen for biomarker discovery. In particular, thanks to the use of FFPE histological sections, a much higher resolution level is reached, which allows an accurate distinction of tumor areas with specific characteristics that otherwise would not be identifiable.

The aim of the present study is to analyze in MIBC the transcriptome of mammary neoplastic epithelium at different stages of progression together with the respective stroma in order to obtain an overview of the temporal modulation of the gene expression profile during tumor progression enriched by the gene expression profile of the stroma surrounding each tumoral portion at each stage.

## Methods

### Tissue samples

FFPE blocks from 7 patients diagnosed with MIBC were selected from the Division of Pathology, Pisa University by senior pathologists. Well recognized and approved guidelines of TNM Staging System [7] were used to select the samples. In particular, the identification of the invasive cancer cell portions was performed by immunohistochemistry with p 63 [8, 9] in order to identify the absence of myoepithelial cells surrounding nests of carcinoma cells [10].

### Laser capture microdissection (LCM) and RNA Extraction

Two mm thick sections were cut from each sample using a new microtome blade for each slide and H&E staining was performed. The PALM RoboMover automatic laser microdissector (Carl Zeiss, Oberkochen, Germany) was used to select the epithelial and stromal cell population. For each sample, seven portions of about 200 cells were microdissected: four related to the breast epithelium and three to the stroma. From the seven tumors we obtained a total of 49 microdissected areas. RNA extraction was performed after an incubation with 50 μl of lyisis buffer PKD (Qiagen, Venlo, Netherlands) and 10 μl of proteinase K at 55 °C over night. The automated system Maxwell 16 (Promega, Madison, WI, USA) using the Maxwell^®^ 16 LEV RNA FFPE Purification Kit was used to perform RNA extraction. As expected, RNA concentration was not measurable because of the low amount of material.

The μm^2^ values of the microdissected areas of the seven samples are shown in Table 1.

**Table 1:** Area of the selected microdissected portions of the seven MIBC samples

Case	NBE	CIS	IBC	EIF	S-NBE	S-IBC	S-EIF
MIBC1	139,575	165,015	93,251	190,167	146,984	190,455	126,573
MIBC2	97,640	140,629	83,000	123,622	154,130	97,545	95,000
MIBC3	198,832	247,052	126,534	187,737	187,680	101,560	196,200
MIBC4	172,604	186,191	182,241	192,628	194,230	116,105	154,600
MIBC5	152,361	195,711	68,696	98,384	155,591	61,656	83,596
MIBC6	165,000	321,680	95,000	166,000	198,000	125,000	182,000
MIBC7	171,000	246,620	101,000	98,000	201,500	80,000	170,000

### cDNA synthesis and amplification

To prepare cDNA from RNA samples, we used the SMARTer Universal Low Input RNA kit (Clontech Laboratories, Takara Bio Inc., Mountain View, CA, USA) that allows high-quality cDNA synthesis starting from as little as 200 pg of input RNA. This kit has been validated for analysis with next-generation sequencing (NGS) instruments to produce NGS-quality cDNA from low concentrations of degraded samples.

### Library preparation and sequencing

To prepare the DNA library we used Nextera XT kit (Illumina, San Diego, CA, USA) following the guidelines of the protocol. We load a maximum of six pooling libraries for each cartridge NextSeq High Output (300 cycles) run on a NextSeq 500 instrument (Illumina, San Diego, CA, USA).

### Data analysis

The data generated by the NextSeq 500, after converting into fastq format with Bcl2toFastq (version 2.17.1.14; Illumina), were mapped against the reference genome (Hg19) by using STAR aligner (version 2.5.3a). The created bam files were then imported into the SeqMonk (version 1.42.0, Babraham Bionformatics), a tool to enable the visualization and analysis of the mapped sequence data. The data were quantified using the RNA-seq pipeline, included in the previous software, and transformed into log2 format. Data intensity filter, included in SeqMonk, was used to highlight differences in gene expression between different portions. Gene expression patterns of the epithelial and stromal portion at each stage of tumoral progression were compared to each other using SeqMonk, setting, when possible, the threshold of the raw *p* value at 0.05 and log2fold at > 2. Dendrograms and Heatmaps were generated with R (version 3.5.1; pheatmap and dplyr libraries), while SparkLine graphs and tables were created with Excel. Furthermore, to summarize high-dimensional gene expression data we used gene set enrichment analysis (GSEA) [11] which is a common approach to interpreting gene expression data based on the functional annotation of the differentially expressed genes. This is useful for finding out if the differentially expressed genes are associated with a certain biological process or molecular function. We used the GSEA tool combined with the interrogation of different gene sets belonging to the molecular signatures database (MsigDB), in particular: the Kyoto Encyclopedia of Genes and Genomes (KEGG), the Hallmarks and Gene Ontology (GO) gene set databases.

## Results

### LCM areas

**Fig. 1:**
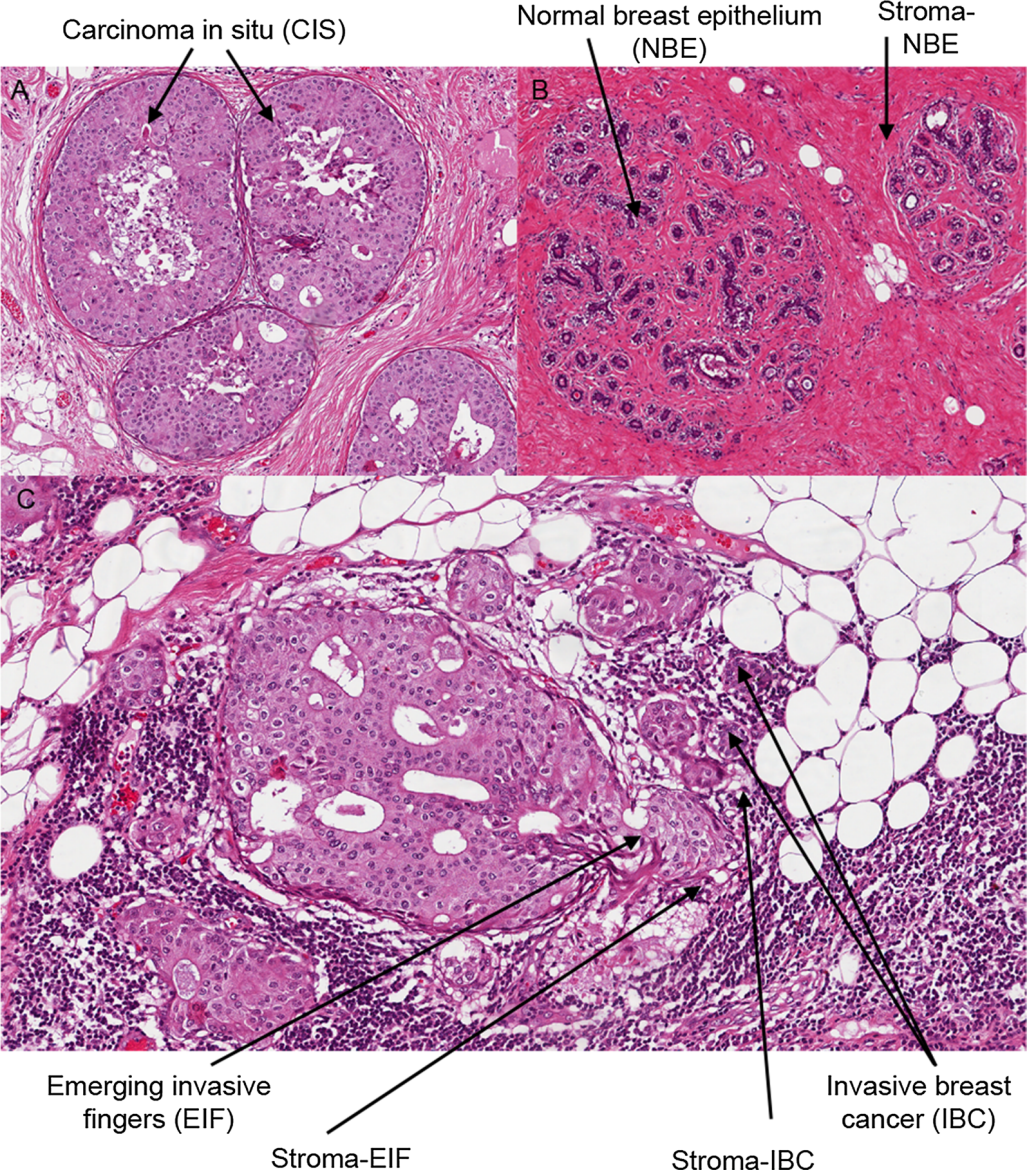
Example of H&E sections of MIBC sample: **A** shows the CIS surrounded by the myoepithelial cells. In **B** the normal portion of the sample is shown, with the NBE and the respective stroma. Instead in **C** the EIF cells with their stroma and the IBC cells with their stroma are shown

Breast epithelium was chronologically divided into normal breast epithelium (NBE), carcinoma in situ (CIS), emerging invasive fingers (EIF) and invasive breast cancer (IBC) (Fig. 1A–C). For each of the breast epithelium portions we collected the adjacent stroma (S) except for the in situ portion: S-NBE, S-EIF and S-IBC (Fig. 1B, C).

### Hierarchical clustering analysis

**Fig. 2:**
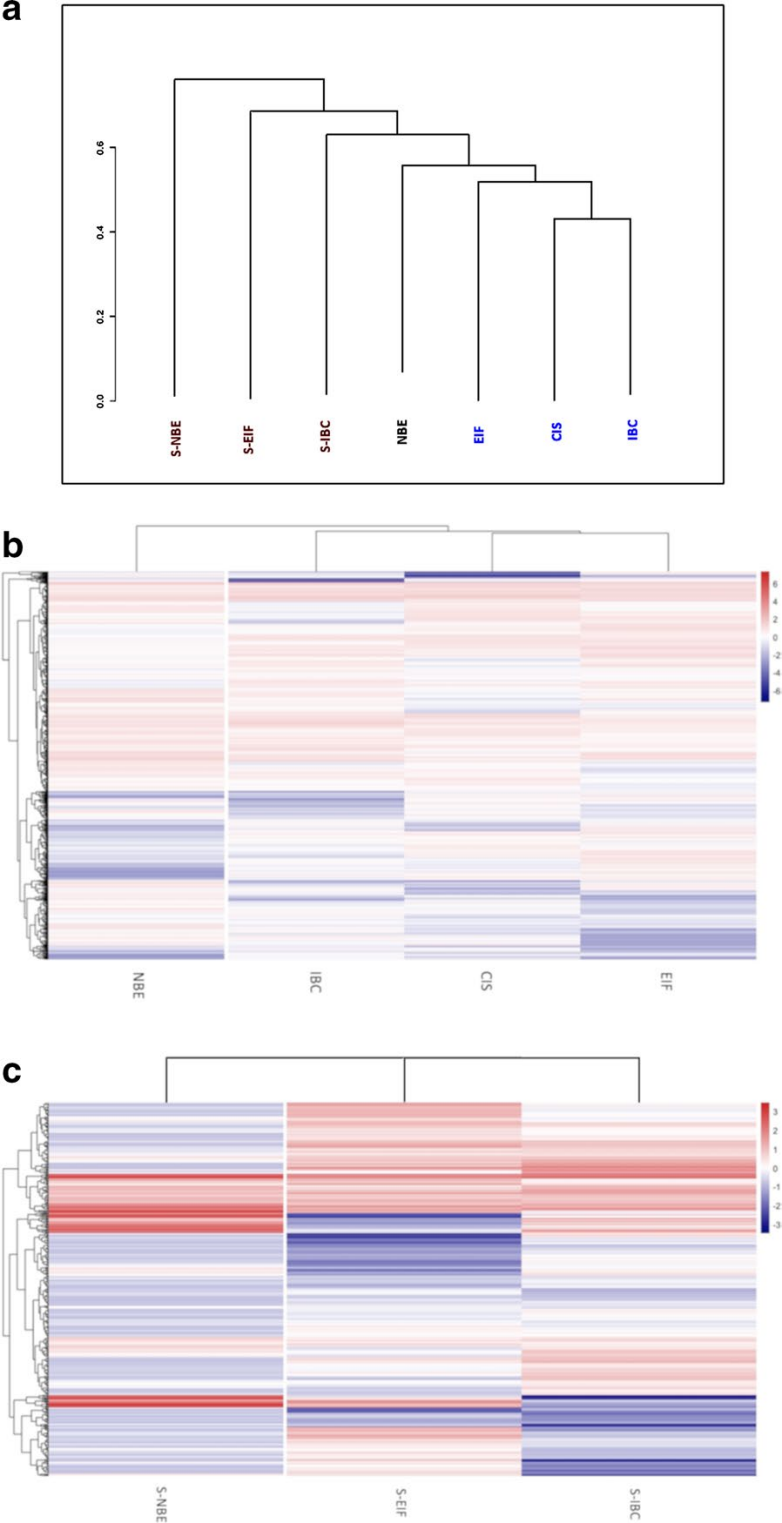
**a** Unsupervised hierarchical clustering made with SeqMonk after transcriptome analysis shows the separation between the stromal and epithelial samples; **b**, **c** overall DEGs of the epithelial and stromal samples respectively were grouped in Heatmaps generated with R software

By deep sequencing of the total RNA, we obtained an average of 24,694,286 reads per sample (ranging from 1,717,350 to 123,193,950) with an average mapping rate of 57% to the reference human genome (hg19). Unsupervised hierarchical clustering analysis generated a dendogram showing a clear distinction between stromal and epithelial samples (Fig. 2a). Moreover in both epithelial and stromal portion the maximum distance, which describes the biggest dissimilarity, is observed, as expected, between the normal and the most advanced stage of cancer progression.

### Identification and pathway analysis of the differentially expressed genes (DEGs)

The overall DEGs deriving from the comparisons within the epithelial and stromal portions were used to display a heatmap in which genes were grouped based on their pattern of gene expression. In Fig. 2b, c the data are displayed in a grid where each row represents a gene and each column represents an epithelial or stromal microdissected portion. The heatmap was combined with a clustering method which group genes and samples together based on the similarity of their gene expression pattern. It is clear that in both Fig. 2b, c, the epithelial and stromal counterparts localize in a separate branch compared to the tumoral portions.

**Fig. 3:**
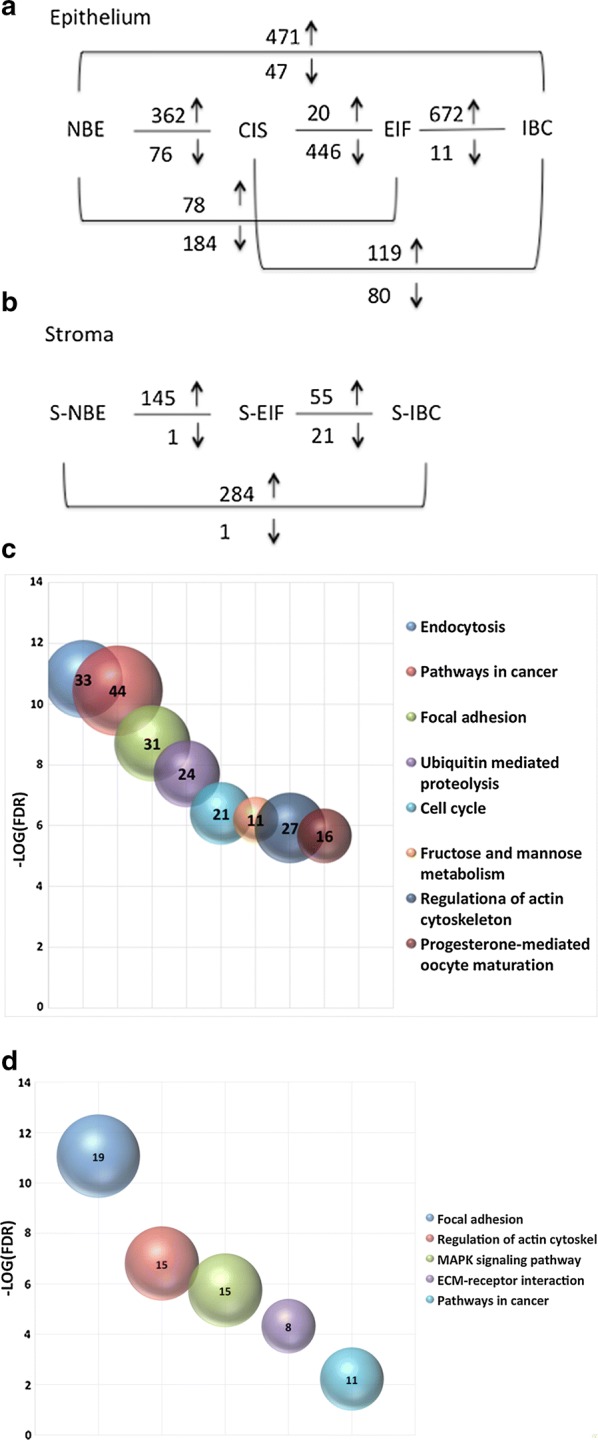
**a**, **b** Upregulated and downregulated DEGs of the epithelium and stromal samples respectively are shown; **c** bubble plots showing the most significant pathways in which the DEGs derived from the epithelium portions comparisons are involved on the basis or the FDR value; **d** bubble plots showing the most significant pathways in which the DEGs derived from the stromal portions comparisons are involved on the basis or the FDR value. Both the bubble plots were generated with Excel based on the results obtained with GSEA tool combined with the KEGG data set

In Fig. 3a, b the upregulated and downregulated DEGs arising from the different comparisons within the epithelium and stromal groups, are shown. The complete list of DEGs derived from these comparisons are listed in Additional file 1: Table S1.

To understand the biological implications, the overall DEGs were investigated using the GSEA tool supported by several molecular signature databases as reported in the materials and methods section. Several signaling pathways (p < 0.05) emerged interrogating the KEGG database. In Fig. 3c, d the most significant pathways are shown according to their FDR value. The size of each circle reflects number of genes included in the pathway and they are ordered according to its FDR value. The pathways obtained from the DEGs derived from the epithelium portions comparisons exploring the KEGG database are the following: endocytosis, pathways in cancer, focal adhesion, ubiquitin mediated proteolysis, cell cycle, fructose and mannose metabolism, regulation of actin cytoskeleton and progesterone-mediated oocyte maturation pathway (Fig. 3c). Equally, the DEGs from stromal portions comparisons, were grouped, according to the KEGG database identifying the following pathways: focal adhesion, regulation of actin cytoskeleton, MAPK signaling pathway, ECM receptor interaction and pathways in cancer (Fig. 3d).

### DEGs obtained from the comparison between tumoral epithelial portions (CIS, EIF, IBC) versus normal epithelial portion (NBE)

**Fig. 4:**
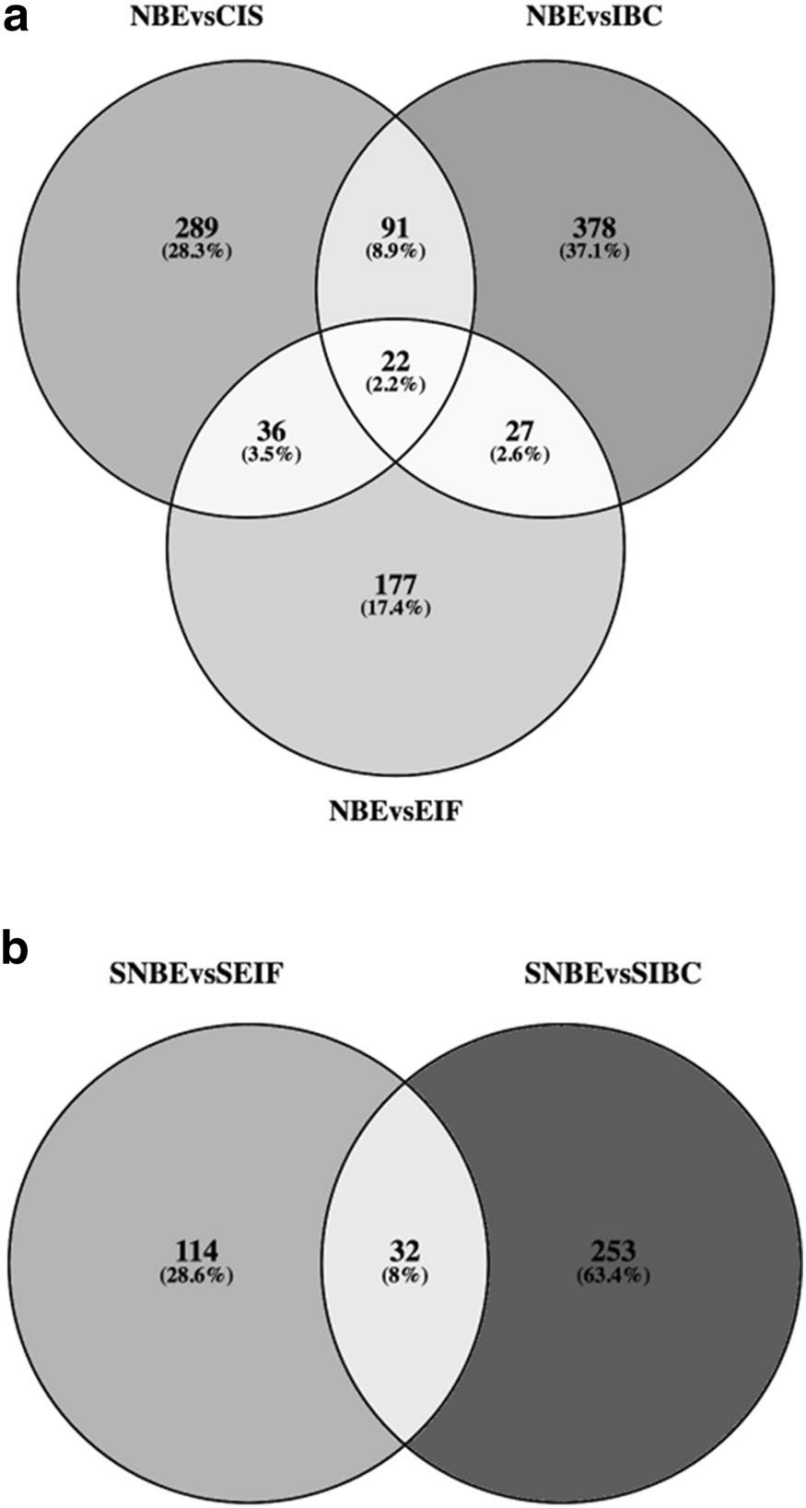
**a** Venn diagram with comparisons between NBE versus CIS, NBE versus EIF and NBE versus IBC; **b** Venn diagram with comparisons between S-NBE versus S-EIF and S-NBE versus S-IBC

After analyzing the overall DEGs, we focused on the DEGs arising from the single comparison between the normal tissue and each distinct tumoral portions of the progression. In Fig. 4a the Venn diagram describes the comparisons between NBE versus CIS, NBE versus EIF and NBE versus IBC. Twenty-two genes are common for all the intersections. The 22 genes are reported in Table 2 with the description of each gene and the gene expression value modulation during tumoral progression from NBE to CIS, EIF and eventually to IBC. Interestingly, the MARS gene has a gene expression that gradually increases with tumoral progression, while FAT2 and CWC15 genes show a gradual decrease.

**Table 2:**
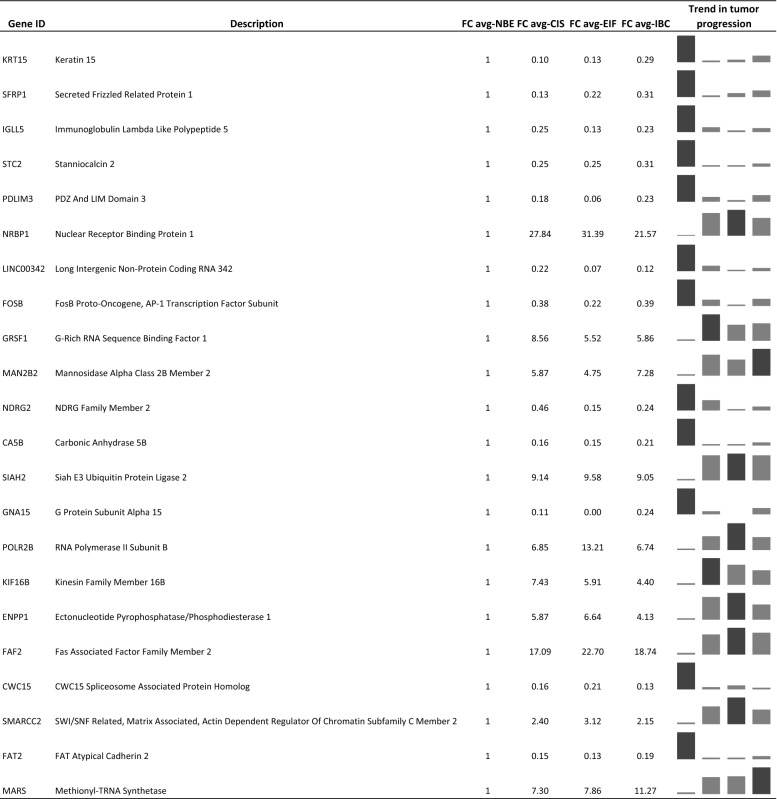
DEGs obtained from the comparison between tumoral epithelial portions (CIS, EIF, IBC) versus normal epithelial portion (NBE) with the respective fold changes values

Sparkline graphs with the fold change value for each gene are shown

All 22 genes were subjected to the GSEA analysis, and the exploration of the Hallmarks gene-set localizes four of them, PDLIM3, SIAH2, STC2 and KRT15 in the early response to estrogen pathway. While, interrogating the GO gene-set, we observed that three genes, SFRP1, KIF16B and POLR2B are involved in the response to fibroblast growth factor pathway.

### DEGs obtained from the comparison between tumor stromal portions (S-EIF and S-IBC) versus normal stromal portion (S-NBE)

Comparing the DEGs obtained from the comparison of S-NBE versus S-EIF and S-IBC we identify 32 genes concurrent in all the intersections (Fig. 4b). In Table 3 we report the 32 genes with their decription and their gene expression values in the different stages of cancer progression from S-NBE to S-EIF and S-IBC. Focusing on the gene expression modulation associated to the cancer progression, we discovered many genes with a gradual increase of expression from S-NBE to S-EIF and to S-IBC, such as KIAA0368, KIAA1217, STAT2, TRAK1, DDX17, IGF2, HIPK3, AQP1, ACADVL, HSPG2, FLNA, NFE2L1, COL1A1, MXRA5, DYSF, SIN3B, JMJD1C and NOTCH2. No gene shows a decreasing downregulation in the progression from S-NBE to S-EIF and S-IBC.

**Table 3:**
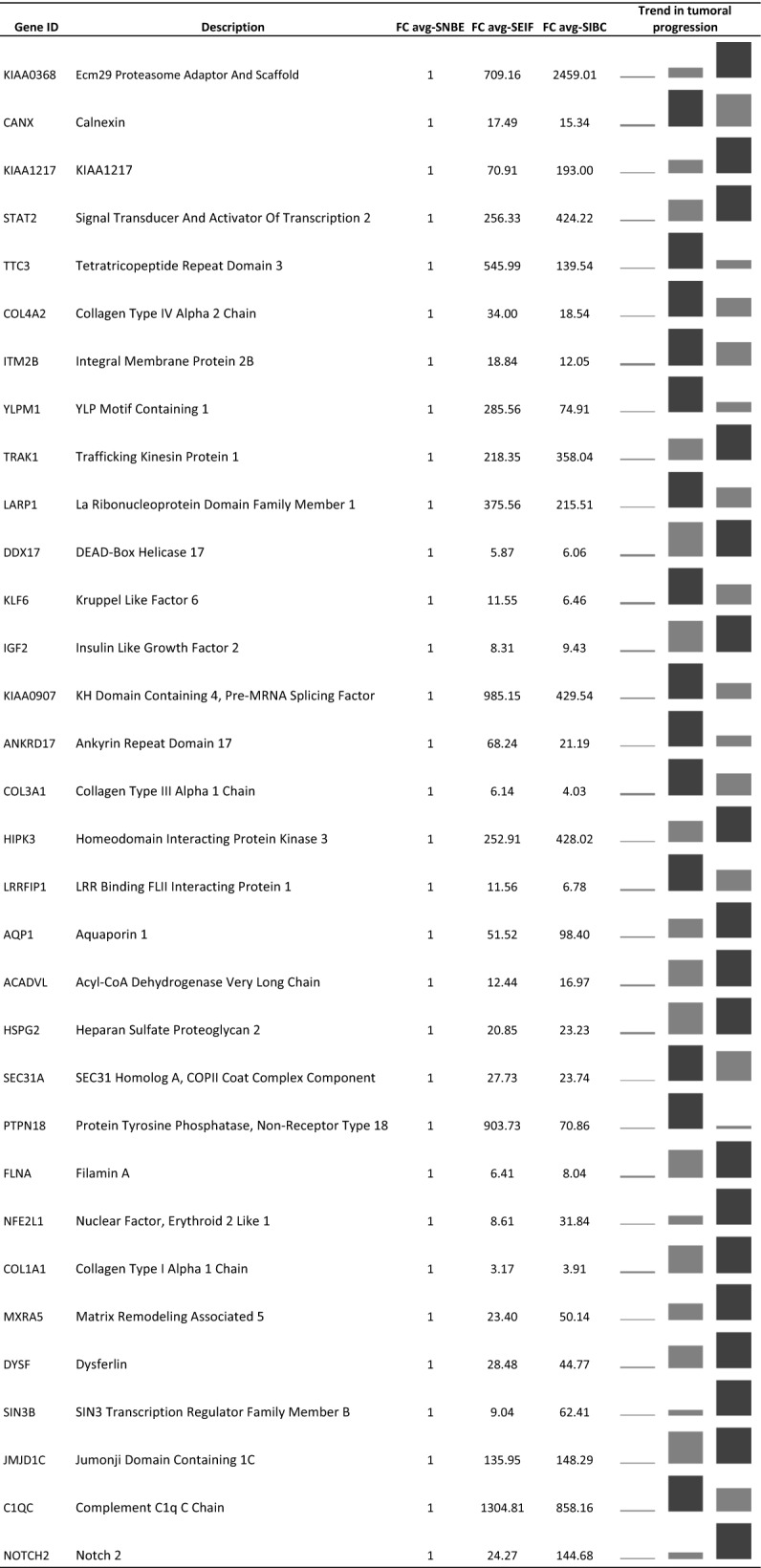
DEGs obtained from the comparison between stromal tumor portions (S-EIF and S-IBC) versus normal stromal portion (S-NBE) with the respective fold change values

Sparkline graphs with the fold change value for each gene are shown

The GSEA tool analysis performed on the 32 genes, revelead, by the Hallmark gene-set, a group of six genes in the epithelial-mesenchymal transition pathway: COL3A1, COL1A1, NOTCH2, COL4A2, FLNA and MXRA5.

Within all stromal portions comparisons, of the 32 genes, three genes were always statistically significant such as NOTCH2, KIAA0368 and NFE2L1, which are shown in Table 4 with the gene description and the fold change expression value. The level of expression for all three genes increases progressively from S-NBE to S-EIF till S-IBC.

**Table 4:**
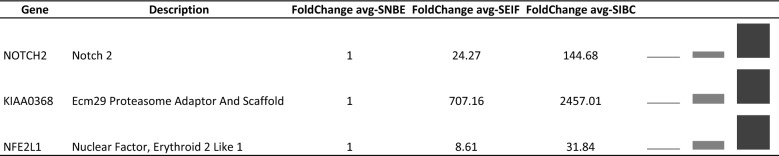
Description of the three genes statistically significant in all the stromal compartments comparisons with the respective fold change values

Sparkline graphs with the fold change value for each gene are shown

### Metabolism pathway analysis: a supervised approach

**Fig. 5:**
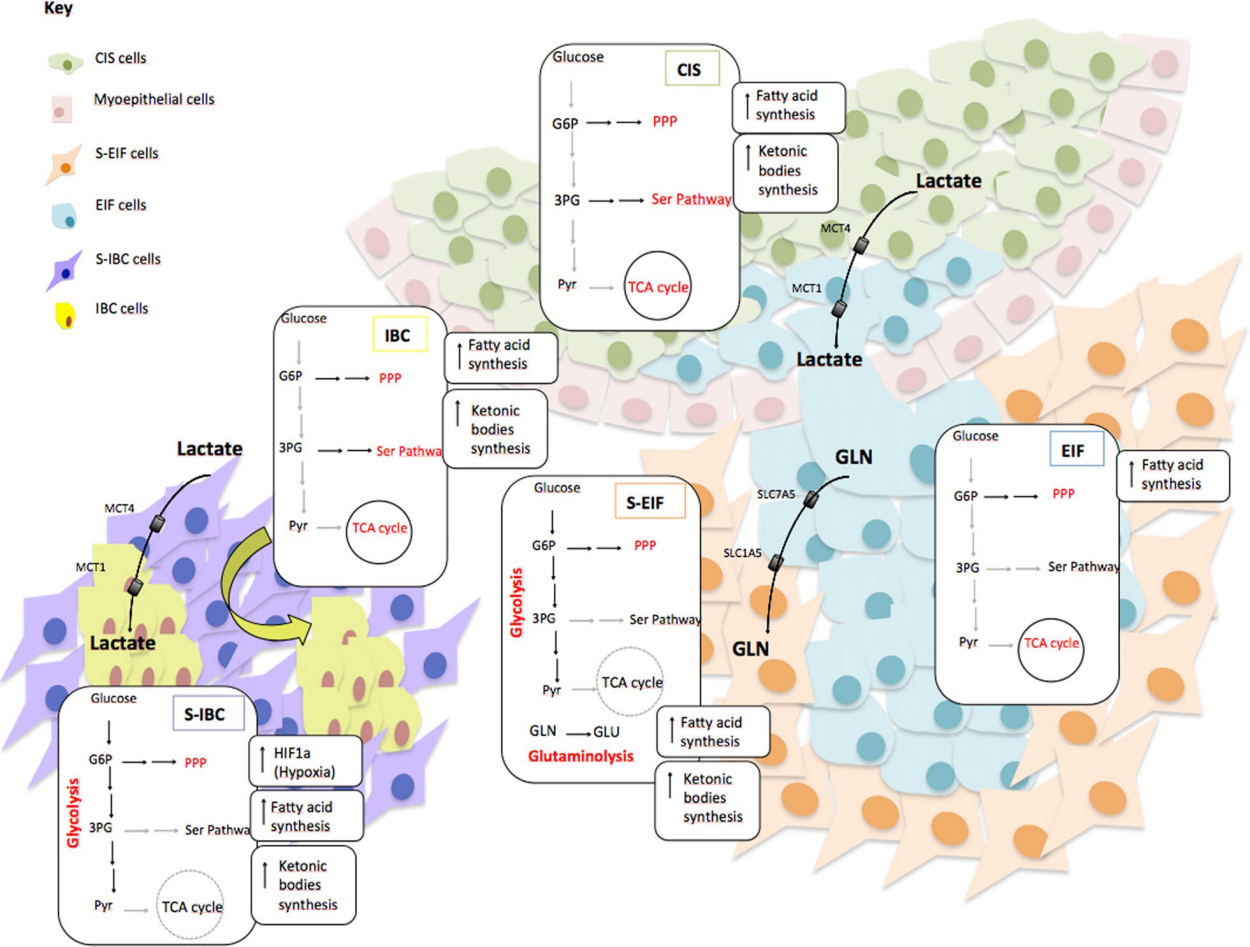
Metabolic pathways and their alterations in the different compartments of MIBCs. In green are shown the CIS cells surrounded by myoepithelial cells (pink cells). In orange, S-EIF cells are shown. These cells surround the EIF cells, in light blue. On the left, IBC cells in yellow and their stromal counterpart, S-IBC in purple

Cancer metabolism is one of the oldest areas of research in cancer biology. The issue is based on the concept that metabolic activities are altered in cancer cells compared to normal cells, and that these alterations support the acquisition and maintenance of malignant properties. Because some altered metabolic characteristics are observed quite generally across many types of cancer cells, reprogrammed metabolism is considered a hallmark of cancer [12]. How metabolism is reprogrammed in cancer cells and how to exploit metabolic changes for therapeutic benefit are among the key questions driving research in the field [12]. Guided by this and thanks to the achievement of a solid transcriptome, describing the variations of genes expression occurring in the single compartments of its microenvironment during MIBC tumor progression, we decided to perform a supervised analysis of all gene expression changes of most specific genes involved in cell metabolism. Therefore we analysed gene expression changes through the different tumor microenvironment portions (compared to NBE and S-NBE) during the different phases of cancer progression, as shown in Fig. 5. Data of selected genes are shown in Additional file 1: Table S2 and Additional file 1: Table S3.

#### CIS microenvironment (green area in Fig. 5)

In the CIS we observe a higher expression of hexokinase 1 (HK1) with consequent high levels of glucose-6-phosphate (GLU6P) derived from glucose, with an activation of the pentose phosphate pathway (PPP) (represented by G6PD and PGD genes) and serine pathway (PSAT1 gene) but without an activation of glycolysis (GPI, LDHA genes). Moreover there is a higher expression of genes linked to ketonic bodies synthesis (HMGCS1 gene), fatty acid synthesis (FASN, ACLY genes) and TCA (tricarboxylic acid) cycle (SDHA, FH genes). We observed, also, an increase in the expression level of MCT4 gene, a carrier that brings the lactate out of the cell. Also the transporter of glutamate (GLU) inside the mitochondrion, SLC25A22 gene, has a higher expression, as a consequence there is a high expression of GLUD2 gene that converts GLU in α-ketoglutarate (α-KG) that enters in TCA cycle. Therefore, in this compartment an oxidative metabolism is detected, with a higher activation of TCA cycle rather than glycolysis.

#### EIF microenvironment (light blue area in Fig. 5)

Also in these cells, glycolysis seems not be appropriately supported; the glucose inside the cells enters in the PPP (G6PD gene). There is a higher expression of the MCT1 gene, the carrier that brings the lactate inside the cell, released by the CIS portion as described above. The fatty acid synthesis is also detected (FASN gene). Besides, as described in the CIS, there is higher expression of SLC25A22 gene and consequent high GLUD2 gene and activation of TCA cycle (SDHA gene). A higher expression (statistically significant) of SLC7A5 gene, the carrier that brings glutamine (GLN) out from the cell, is also observed. In conclusion, it seems that also this compartment is characterized by an oxidative metabolism.

#### S-EIF microenvironment (orange area in Fig. 5)

In the S-EIF, there is a greater activation of glycolysis (GPI, LDHA genes). There is higher expression of MCT1 gene, carrying the lactate inside the cell and it is converted in PYR (Pyruvate) due to the high expression of lactate dehydrogenase B (LDHB) gene. Moreover we see the activation of the glutaminolysis: a high expression of SLC1A5 gene, the carrier that brings GLN inside the cell, which seems to be released by the EIF portion. Then GLN is converted in GLU and brought in the mitochondrion (higher expression of SLC25A22 gene) where is converted in α-KG. However, TCA cycle is not very triggered (CS, OGDH, SDHA genes). PPP, fatty acid synthesis and ketonic bodies synthesis are observed. So, in the S-EIF portion, we note a glycolytic metabolism and moreover a higher production of different energy sources (represented by fatty acids and ketonic bodies).

#### IBC microenvironment (yellow area in Fig. 5)

In these cells there is glucose that enters the cell (GLUT1 gene). Also in these cells, like in CIS and EIF, the glucose is not involved so much in glycolysis but in the PPP (G6PD gene) and Serine pathway (PSAT1 gene). Moreover there is a high level of fatty acid synthesis (FASN gene) and ketonic bodies synthesis (HMGCS1 gene). TCA cycle is also detected (SDHA, SL25A22 genes). There is, also, high quantity of lactate entering the cell because of higher expression of the MCT1 gene. Therefore also this compartment shows a type of oxidative metabolism, just like in CIS and EIF compartments.

#### S-IBC microenvironment (purple area in Fig. 5)

The expression of HIF1α gene, responsible of hypoxia, is statistically higher in in this compartment. Glycolysis is activated (GPI gene) and there is higher expression of MCT4 gene, the carrier that brings lactate outside the cell. There is a high overflow of GLN outside the cell, due to high levels of SLC7A5 gene. PPP (PGLS gene), fatty acid synthesis (ACLY gene) and ketonic bodies synthesis (ACAT1, BDH1 genes) are observed. Instead TCA cycle is not well activated.

## Discussion

Breast cancer is the most common malignancy and the leading cause of cancer-related death in women worldwide. The microenvironment of these cancers is now recognized as a critical participant in tumor progression. Recent data demonstrate significant gene expression in cells composing the microenvironment during disease progression, which can be explored as biomarkers and targets for therapy. Indeed, gene expression signatures derived from tumor stroma have been linked to clinical outcomes. The tumor microenvironment has assumed a progressively increasing importance over the years; infact a continuos interaction is obtained: on one hand, the tumor is able to influence the microenvironment thanks to extracellular signals, promoting phenomena such as neoangiogenesis and immuno-tolerance; on the other, the cells of the microenvironment favor tumor progression. There is increasing interest in refining our current understanding of the tumor microenvironment. An in-depth study of the tumor microenvironment, can provide information on both the molecular mechanisms underlying the progression as well as on possible etiological factors. In fact, except for some hypotheses of viral etiology [13], we are not yet aware of the etiological cause of breast cancer.

The aim of this study was to analyze the gene expression pattern of microdissected tumoral epithelial cell areas related to each phase of tumoral progression in breast cancer (CIS, EIF and IBC) compared to the normal epithelial cells area (NBE). At the same time we studied also the stromal portions around the tumoral epithelial areas (S-EIF and S-IBC) in comparison to the stromal area surrounding the normal epithelial mammary tissue (S-NBE). We decided to collect these areas, respectively, from 7 patients utilizing the MIBC type, in which cancer progression phases are still very distinguishable. This approach has the advantage of giving a more integral view of the transcriptome changes occurring during cancer progression and allows the investigation of interactions between compartments. The approach can also give insights on the molecular mechanisms that govern cell–cell interactions.

From all gene expression level comparisons, some key aspects have emerged. Focusing on the overall DEGs in epithelial portions, the main pathways in which DEGs were grouped are the endocytosis process, the pathways in cancer and interestingly the fructose and mannose metabolism. Cancer metabolism is essential for the maintenance of cell proliferation in a tumor. The pioneering studies of O. Warburg [14] asserted that a cancer cell needs an increase in glycolysis and a decrease of oxidative metabolism. Nowadays, after several further investigations, the starting concept has been revisited. Metabolism heterogeneity is well known in cancer, both for cancer cells and for the cells of the microenvironment. So a single metabolic program can not be representative of the global metabolism of a tumor. Infact, fructose metabolism, for instance, is different from that of glucose. Through the PPP, fructose induces NADPH and nucleotides synthesis. Besides, glucose also generates fructose through a specific pathway, the polyol pathway, and some of its metabolites (ex. glycolaldehyde and glyoxal) can affect cell survival [15]. Through this mechanism, this type of metabolism can have a role in neoplastic growth.

The overall DEGs identified in stromal samples, are grouped essentially into the focal adhesion process, the extracellular matrix (ECM) receptor interaction pathway and regulation of actin cytoskeleton pathway. All these processes are linked to cell motility, essential for invasion and for metastasis formation. Cancer cell movement during invasion is a complex system made mainly of membrane protrusions (lamellipodia) arising at the leading edge of migrating cancer cells after activation by extracellular stimuli. Afterwards, the leading membrane is fixed by nascent sites of attachment (focal adhesions) [16]. The F-actin stress fibres contract, creating the tension needed to drag the cell forward, with loss of adhesion at the rear of the cell, so the cell retracts and is dragged in the direction of migration [17]. The involvement, that we detected, of these pathways in the stroma-derived samples is, therefore, perfectly in agreement with the literature, since in our study tumor epithelial cells are progressing towards a real invasion supported by the stromal cells in the process of tumoral progression.

Among all DEGs derived from the comparisons done within tumoral epithelial samples, we identified some key genes that gradually decrease or increase their expression with tumoral progression: KRT15, SFRP1 and MARS. KRT15 (Cytokeratin 15) is a cytoskeletal protein, expressed essentially in the epithelial cells and considered a marker of epithelial stem cells [18]. In our samples we observed a significant decrease of expression in tumoral cells compared to the normal tissue, this is in accordance with Shen et al. in a study on esophageal squamous carcinoma [19] even if in literature there are conflicting results about its role in cancer [20–22]. SFRP1 (Secreted Frizzled Related Protein 1) is a member of SFRP family whose function is to modulate Wnt signaling through direct interaction with Wnts. This gene has already been found involved in breast cancer tumor progression as a tumor suppressor gene and moreover it has been proposed as a target gene for early diagnosis [23]. In our samples SFRP1 expression levels are in complete accordance with the literature, with a decrease during tumoral progression [23]. MARS (Methionyl-TRNA Synthetase) is a member of the class I family of aminoacyl-tRNA synthetases. We found this gene with a gradual increase of expression in tumoral portions, in accordance with the paper of Kim et al. [24] that observed MARS overexpression in non-small cell lung cancer, associated also with a poor prognosis.

The key genes identified among DEGs obtained from the comparisons within the stromal portions are: STAT2, NFE2L1, SIN3B and NOTCH2. All these genes showed a gradual upregulation during the tumoral progression. STAT2 (signal transducer and activator of transcription 2) is a member of STAT family proteins generally involved in response to interferon. In particular STAT2 is a necessary transcription factor in the IFN-α/β signaling pathway [25]. Ogony et al. [26] studied STAT2 in breast cancer cells as a key regulator of the expression of IFITM1 (interferon-induced transmembrane protein 1); together they are involved in the IFNα signaling pathway, in particular their overexpression promote cancer aggressiveness in breast cancer, that agrees with our data. Moreover in literature some papers are already reporting data about IFNα immunotherapy and STAT2 status in melanoma [27] and in other type of diseases [28]. NFE2L1 (nuclear factor, erythroid 2 like 1) is a protein that is involved in globin gene expression in erythrocytes, this protein is not yet well studied, the most important function seems to be related to proteasome process [29]. Very different it is the case of SIN3B (SIN3 transcription regulator family member B), a well known protein that interacts with MYC (MYC proto-oncogene, BHLH transcription factor), which was observed promoting cancer progression and metastasis in breast cancer [30] in accordance with our data. Also NOTCH2 (neurogenic locus notch homolog protein 2) is a very well known protein, that functions as a receptor for membrane-bound ligands jagged-1 (JAG1), jagged-2 (JAG2) and delta-1 (DLL1) to regulate cell-fate determination. Several studies have been conducted on NOTCH2 and cancer, not all in accordance with our results. Some studies describe NOTCH2 as a tumor suppressor gene in breast cancer [31, 32] while as an oncogene in bladder cancer [33] promoting cancer growth and metastasis through epithelial–mesenchymal transition (EMT), which process is fully consistent with our findings. It is important to point out that this is the first time that STAT2, NFE2L1, SIN3B and NOTCH2 genes are described associated to the cancer stroma.

Because of the heterogeneity of cancer cells, each tumor differs in its metabolic status [34]. This is well demonstrated in our samples. In detail, we can deduce that the CIS is a so-called oxidative tumor, because no glycolysis is activated, but there is a great activation of TCA cycle from which the cell receives the energy. This is in accordance with some studies demonstrating that there are tumors, such as the oxidative tumors, where glycolysis is not predominant [35]. Furthermore, we observed in the CIS compartment, a release of lactate from the cell due to an upregulation of the MCT4 gene. We can assume, therefore, that the lactate, released by CIS, enters in EIF cells, which present an upregulation of the MCT1 gene. EIF cells, which are about to invade, like CIS cells, have a lower activation of glycolysis in favor of the TCA cycle. Also these tumoral cells behave like oxidative tumor cells. Moreover, in the EIF cells, a release of GLN is detected, which enters in the surrounding S-EIF cells and is used as energy fuel, generating GLU through the glutaminolysis. According to our observations, the S-EIF compartment undergo aerobic glycolysis and generate high levels of fuels like fatty acids, lactate, ketonic bodies in compliance to what the reverse Warburg effect describes. It is well known, infact, that in the reverse Warburg effect, CAFs “feed” the tumoral cells with glycolysis and fatty acid and ketonic bodies synthesis [36, 37]. In turn, cancer cells produce ATP through the TCA cycle and mitochondrial oxidative phosphorylation system (OXPHOS) [38, 39], as we observed in EIF cells. When we focus on the invasion process, the IBC cells show a similar reverse Warburg metabolic situation as in the EIF cells. S-IBC cells are, indeed, characterized by a glycolytic metabolism with release of lactate that enters the IBC tumoral cells, which show an oxidative metabolism.

## Conclusions

Our data describe, by the use of LCM on FFPE tissues, the changes of gene expression values during cancer progression in the epithelial cells enriched by the gene expression changes of the surrounding stromal cells. This is the first time that such gene expression values are obtained from FFPE microdissected areas localized on the same tissue section. It is well known that CAFs are necessary for the development and the maintenance of the tumor and particularly for tumor progression. Since we are facing a new phase where there is a conversion from a cancer cell-centric strategy to a stroma-centric strategy, it is crucial to pursue further investigations to better clarify the role of CAFs in tumor progression.

## Additional files

**Additional file 1.** Complete list of DEGs derived from all the comparisons: S-NBE versus S-EIF, S-NBE versus S-IBC and S-NBE versus S-EIF.

**Additional file 2.** List of observed genes in the epithelial portions involved in the metabolism. The fold change values comparing NBE and tumor portions are reported.

**Additional file 3.** List of genes observed in the stromal portions involved in the metabolism. The fold change values comparing S-NBE versus S-EIF and S-NBE versus S-IBC are reported.

## Data Availability

All data generated or analyzed during this study are included in this published article.

## Abbreviations

DCIS: ductal carcinoma in situ
CAFs: cancer associated fibroblasts
MIBC: microinvasive breast carcinoma
FFPE: formalin-fixed, paraffin-embedded
LCM: laser capture microdissection
NGS: next-generation sequencing
GSEA: gene set enrichment analysis
MsigDB: molecular signatures database
KEGG: Kyoto encyclopedia of genes and genomes
GO: gene ontology
NBE: normal breast epithelium
CIS: carcinoma in situ
EIF: emerging invasive fingers
IBC: invasive breast cancer
S-NBE: stroma-normal breast epithelium
S-EIF: stroma-emerging invasive fingers
S-IBC: stroma-invasive breast cancer
DEGs: differentially expressed genes
HK1: hexokinase 1
GLU6P: glucose-6-phosphate
PPP: pentose phosphate pathway
TCA: tricarboxcylic acid
GLU: glutamate
α-KG: α-ketoglutarate
GLN: glutamine
PYR: pyruvate
ECM: extracellular matrix
KRT15: cytokeratin 15
SFRP1: secreted frizzled related protein 1
MARS: methionyl-TRNA synthetase
STAT2: signal transducer and activator of transcription 2
IFITM1: interferon-induced transmembrane protein 1
NFE2L1: nuclear factor, erythroid 2 like 1
SIN3B: SIN3 transcription regulator family member B
MYC: MYC proto-oncogene, BHLH transcription factor
NOTCH2: neurogenic locus notch homolog protein 2
JAG1: jagged-1
JAG2: jagged-2
DLL1: delta-1
OXPHOS: oxidative phosphorylation system
